# Factor structure and psychometric properties of Polish version of Parental Bonding Instrument (PBI) among adults and adolescents

**DOI:** 10.1371/journal.pone.0272617

**Published:** 2022-08-25

**Authors:** Zbigniew Wajda, Bernadetta Izydorczyk, Katarzyna Sitnik-Warchulska, Sebastian Lizińczyk, Jakub Lickiewicz

**Affiliations:** 1 Faculty of Management and Social Communication, Institute of Applied Psychology, Jagiellonian University, Krakow, Poland; 2 Faculty of Philosophy, Institute of Psychology, Jagiellonian University, Krakow, Poland; 3 Faculty of Psychology, SWPS University of Social Sciences and Humanities, Katowice, Poland; 4 Faculty of Health Sciences, Jagiellonian University Medical College, Krakow, Poland; Tabriz University of Medical Sciences, ISLAMIC REPUBLIC OF IRAN

## Abstract

Parental Bonding Instrument (PBI) by Parker et al., is a widely known and used tool in studies on the assessment of parenting behavior in adult, adolescent and child populations. This tool has had many translations and adaptations globally. In Poland, the factor structure and psychometric properties of PBI have not been studied so far. The aim of the presented research was to perform such an analysis both in the group of adults and adolescents. The data from four research projects, in which the 25-item version of the PBI translated into Polish was used, were analyzed. Data from 698 participants in total, including 473 adults and 225 adolescents were collected. Exploratory factor analyzes was performed for both mother and father version. A study of the reliability of individual factors, stability over time (test-retest) and an analysis of criterion validity were carried out. Both in the group of adults and adolescents, obtained a three-factor structure, acceptable reliability and stability over time. Moreover PBI correlated with another Polish tool in line with the adopted hypotheses, showing satisfactory criteria validity.

## Introduction

The parent-child relationship is one of the most explored areas in psychology and psychiatry. Research has shown a correlation of properly formed bonding and positive vs. negative parenting practices on the child’s psychological traits (e.g. [1, 2]), social functioning (e.g. [3–5]), as well as mental and somatic health [6–8].

Studying the relationship of younger children with their parents is usually carried out using observational methods, while in older children and adults, self-reporting methods are most often used. One of the best-known tools of this type is the Parental Bonding Instrument (PBI) created by Parker et al., [9], which is intended to investigate retrospective perception of parental behavior that influenced bonding with the child. The examined person separately describes the behavior of the mother and father towards each other in the first 16 years of life. The questionnaire contains 25 identical statements (items) for each of the parents, towards whom the respondent responds on a 4-point scale: very like, moderately like, moderately unlike, very unlike. The authors of the test [9] in the original version identified two dimensions: Care and Overprotection, which allow for the selection of four parental bonding quadrants: "optimal parenting" in other words: high care and low protection, "Affectionate constraint", which means high care and high protection, “Affectionless control” which means high protection and low care and “neglectful parenting” which means low care and low protection.

PBI has become one of the most popular tools to study retrospective perception of parental attitudes, and the basic hypothesis of this model assuming that a high level of care and a less protection are associated with better functioning [9, 10]. Many studies have shown that high care and less protection correlate with mental health and better functioning. For example, in large studies in the USA [11], the Netherlands [12] and other European countries: Germany, France, Belgium and Italy [7], high care and less protection inversely correlated with various types of anxiety disorders, PTSD, personality disorders and addictions. In the studies of Avagianou and Zafiropoulou [13], optimal parental bonding correlated with a lower intensity of depression; in Wajda [14] studies reveal a lower intensity of psychopathology among young girls. According to the systematical review by Tetley et al., [15] lower parental care and higher parental protection occurred in women with eating disorders (this differentiated the group with eating disorders from the control group). Weaker associations between PBI scores and psychopathology were noticed in patients with disorders with a larger biological and psychiatric component (e.g. schizophrenia, endogenous depression, etc.) [16].

More recent PBI research shows that neglectful parenting style perceived by the adolescent and the father as well, characterized the families of patients with internalizing symptoms and also in the families with externalizing adolescents, it was mainly the mother to remember an affectionless control parental style [17]. An inverse relationship between maternal and paternal care and antisocial traits was noted [18], and parental bonding was one of the variables modulated the psychological status during the lockdown [19].

In addition, it should be mentioned that this tool has also confirmed its usefulness in studies of children [20] and adolescents [21]—in this case, the retrospective assessment is not tested but assessed current perception of parent-child bonds. For example, Stein et al., [22] used PBI in a group of children aged 7–16, where children diagnosed with depression reported significantly elevated maternal overprotection and in turn current maternal depression had a deleterious effect on the child’s perception of maternal protection and paternal care. In the studies of Sideridis & Kafetsios [23] conducted among children from elementary school, the results indicated that maternal caring scores were associated with lower levels of fear of failure, anxiety and depression. Cross-sectional studies in children and adolescent have found that PBI indices of negative parenting are associated with poor interpersonal functioning [24], different kind of psychopathology [17, 25, 26] and even prospective research has demonstrated that perception of negative parenting predicts emotional and behavioral dysregulation [27, 28].

The validation process in different countries revealed some differences and inaccuracies. A large leaf of studies from various countries confirmed the two-dimensional construction of PBI, e.g. in Australia [9], in the United Kingdom [29], in Japan [30], in Italy [31], in Turkey [32], in Pakistan [33]. However, it turned out that in Western European countries, the model most often adopts a three-factor structure [34, 35], where research, apart from those distinguished by Parker et al. [9] Care and Overprotection dimensions—showed a third dimension called Autonomy or Encouragement of behavioral freedom. Thus, the 3-dimensional structure was obtained, among others, by: the Spanish version [36], the British version [37, 38], the US [34, 37] French [39], Netherlands [40]. At the same time, the 3-dimensional structure was also obtained from versions outside of Western culture [33, 41].

Other researchers pointed to the 4-dimensional model in Eastern culture countries: in Japan [42, 43], in China [21, 44] in Iran—Persian version [45], and the fourth factor was named Indifference.

It should be remembered that in addition to the above-mentioned differences, there are also other ones, e.g.

- studies were conducted with the use of various versions of the PBI, including: the original, 25-item [9, 46], shortened 16-item [40, 43, 47] and even very short 8-item [35];
- studies showed a structure different in terms of the number of factors, even within the same culture, e.g. in Australia (2-factor: Parker et al. [9] and 3-factor: Cubis et al. [48]) or in Japan (2-factor: Kitamura and Suzuki [30]; and 4-factor: Uji et al. [42]; Suzuki, et al. [43]);
- studies have been conducted in nonclinical sample (e.g. Behzadi and Parker [45]) or in clinical sample (e.g. Kullberg et al. [40]);
- statistical analyzes used confirmatory factor analysis or/and exploratory factor analysis;
- studies were conducted in various age groups: mostly among adults (e.g. [38, 40]) but also among adolescents 11–21 age [21] and even among children 7–12 age [20].

PBI is significantly use globally and also in Poland it was used many times in studies of various groups, e.g. patients with schizophrenia [49], with eating disorders [50], among adolescents with behavioral disorders [51, 52], late adolescents in nonclinical sample [14], adults in nonclinical sample [53], among adolescents with trauma [54] or patients participating in group psychotherapy [55] [NO_PRINTED_FORM]. Despite numerous studies with the use of PBI, the psychometric properties of the Polish version of the PBI have not been investigated so far. All the above-cited studies used the original 25-item version translated into Polish, and the authors followed Parker et al., [9] a two-factor structure with dimensions: care and overprotection. Therefore, it was decided to analyze the Polish version of the PBI questionnaire, which will answer the questions about the number of factors, internal consistency and test-retest reliability, both in the group of adults and adolescents and also criteria validity in group of adults.

## Materials and methods

The study was conducted in accordance with the Declaration of Helsinki, and the protocol was approved by the Ethics Committee of Institute of Applied Psychology, Jagiellonian University in Krakow (79/2021). All participants were informed about its purpose and terms. Written informed consent has been obtained from all participants. The data has been submitted to the Repository of the Jagiellonian University and is available under DOI number: https://doi.org/10.26106/26pj-kc03.

### Study design and participants

The study involved 733 people, but after the rejection of incomplete questionnaires, a total of 698 people were included in the analysis, including 473 adults(men = 177, women = 296; mean age M = 28.59; ME = 25, min-max = 20–55) and 225 people in adolescence (boys = 16, girls = 209; mean age M = 16.00; ME = 16, min-max = 13–19).

The data comes from 4 research projects:

- research conducted as part of a doctoral dissertation, where patients of the day care unit with diagnosis of neurotic, stress-related and somatoform disorders (61%), eating disorders (15%), anorexia nervosa, bulimia nervosa, overeating associated with other psychological disturbances and specific personality disorders (24%) n = 144, (men = 58, women = 86, average age M = 32.51, min-max = 20–54) [56];
- studies conducted as part of two master’s theses involving adults from Southern Poland (city with more than 750.000 habitants), who agreed to participate in the study, without eating disorder diagnosis -n = 130 (men = 65, women = 65, mean age M = 25.45, min-max = 20–36) [57] and adolescent girls from Southern Poland (city with more than 750.000 habitants) (12–18 years) with a diagnosis of the eating disorders (clinical group) and girls in the same age as control group. n = 134 (mean age M = 14.92, max-min = 13–17) [58];
- studies in a group of people in late adolescence, habitants of 150.000 city, who agreed to participate in the study n = 91 (boys = 16, girls = 75, mean age M = 17.91 min-max = 17–19) [14];
- research especially planned and conducted to determination of psychometric properties of PBI, in the group of adults, mainly students as well as participants of workshop and postgraduate studies at few universities in Southern Poland, n = 199 (men = 54, women = 145; mean age M = 27.70; max-min = 20–55) with repeated measurement after 3 weeks, where the second part for mothers was completed by 116 people, and the part for fathers was filled by 112 people. In this research we also used Retrospective Assessment of Parents’ Attitudes Inventory (RAPAI) by Plopa to test the criterion validity of Parental Bonding Instrument.

All questionnaires were filled in by the participants as the paper-pencil version and depending on the above-mentioned research project, they were filled in at the mental health center or university office.

### Measures

a) Parental Bonding Instrument (PBI)—questionnaire by Parker et al. [9, 59] in its original version contains 25 items which are rated using a 4-point, ordered–categorical, Likert-type response scale with a marked tendency toward abnormality and allows the retrospective perception of dyadic ties with parents to be examined. In the original version by Parker et al., [9] showed a two-dimensional structure with Care and Overprotection dimensions. The original dimension of care can range from emotional coldness and rejection, to the emotional warmth, while the overprotection can take the items from the psychological control to psychological autonomy. In this model it is assumed that the most optimal for child development is a relationship with caregiver in which the parent object has a high degree of care and low degree of overprotection.

#### Translation of PBI

The PBI questionnaire was translated into Polish by Agnieszka Popiel and Monika Sitarz, and then back translated by a native American English speaker, both versions were compared by researchers and no significant differences were found. First time in Poland, the tool was used in the study of family relationships in people with schizophrenia by Popiel and Pragłowska [49].

b) Retrospective Assessment of Parents’ Attitudes Inventory (RAPAI) by Plopa [60] was used to test the criterion validity of PBI The measure consists of two questionnaires–to assess mother and father attitudes separately (consist 50 items each of them). It allows to conduct retrospective assessment of parents in terms of five parental dimension (10 items for each dimension):
  - Acceptance–Rejection Attitude dimension (Cronbach’s alpha: for mother = 0,90; for father = 0,89). High results indicate close emotional parent-child relation; low results indicate lack of acceptance (rejection attitude);
  - Demanding Attitude dimension (Cronbach’s alpha: for mother = 0,90; for father = 0,90). High results indicate over-demanding attitude; low results indicate appropriate attitude;
  - Autonomy Attitude dimension (Cronbach’s alpha: for mother = 0,86; for father = 0,87), High results indicate highly desired treatment of the child by the parent and understanding of child’s independence; low results indicate lack of autonomy;
  - Inconsequence Attitude dimension (Cronbach’s alpha: for mother = 0,93; for father = 0,87) high results indicate inappropriate attitude; low results indicate appropriate attitude;
  - Protection Attitude dimension (Cronbach’s alpha: for mother = 0,87; for father = 0,84). High results indicate overprotective attitude; low results indicate appropriate attitude.

### Statistical analysis

In order to examine the psychometric properties of Polish version PBI—exploratory factor analyses was performed for both mother and father version. Afterwards, the reliability coefficients were calculated. All analyses were performed using two statistical programs: Statistica 10.0 and SPSS for Windows 23.0 Whole of this analysis performed for both adults and adolescent. It was included items that loaded more than 0.4 to maximize the factors’ internal consistency—it is one of the most widely utilized approach based on cutoff according to liberal-to-conservative continuum, setting the cutoff at 0.4. [61]. Then, the retest reliability test was carried out for the results of the test repeated after 3 weeks, where 116 people completed the part for mothers and 112 people for fathers. In the last step, an analysis of the correlation between PBI and the Retrospective Assessment of Parents’ Attitudes Inventory (RAPAI) by Plopa [60] was performed to test the criterion validity.

## Results

### Results of exploratory factor analysis in group of adults

Table 1. Shows results of exploratory factor analysis in the group of adults, which showed the three-way structure of the PBI. All items, both in the version for the mother and in the version for the father, exceeded the accepted threshold value of 0.4. In the version for the mother, they loaded the weakest item 6 (Factor 1) and item 25 (Factor 3), and the strongest load item 12 (Factor 1). In the father version, higher loading rates were shown, with item 7 (Factor 2) being the weakest, and item 3 (Factor 3) and item 12 (Factor 1) the strongest.

**Table 1:** Factor loadings of the Polish version of the Parental Bonding Instrument–results of exploratory factor analysis in a group of adults (n = 473).

PBI Items	Factors					
	PBI Mother version			PBI Father version		
	Factor 1- care	Factor 2- overprotection	Factor 3- autonomy	Factor 1- care	Factor 2- overprotection	Factor 3- autonomy
1. Spoke to me in a warm and friendly voice	**0.816**	-0.123	-0.183	**0.804**	-0.122	-0.174
2. Did not help me as much as I needed	**-0.746**	-0.151	0.063	**-0.619**	-0.164	0.025
3. Let me do those things I liked doing	0.011	-0.065	**0.790**	0.094	-0.109	**0.836**
4. Seemed emotionally cold to me	**-0.864**	0.090	-0.154	**-0.804**	0.117	-0.067
5. Appeared to understand my problems and worries	**0.764**	-0.186	0.268	**0.800**	-0.056	0.155
6. Was affectionate to me	**0.474**	0.305	0.245	**0.587**	0.531	0.063
7. Liked me to make my own decisions	0.322	**-0.489**	0.432	0.273	**-0.541**	0.436
8. Did not want me to grow up	-0.225	**0.736**	-0.083	-0.084	**0.804**	-0.079
9. Tried to control everything I did	-0.210	**0.606**	-0.451	0.043	**0.693**	-0.426
10. Invaded my privacy	-0.301	**0.558**	-0.411	-0.108	**0.713**	-0.411
11. Enjoyed talking things over with me	**0.834**	0.020	0.182	**0.824**	0.002	0.111
12. Frequently smiled at me	**0.877**	0.116	-0.180	**0.833**	0.031	-0.119
13. Tended to baby me	0.097	**0.754**	0.081	0.034	**0.603**	0.133
14. Did not seem to understand what I needed or wanted	**-0.662**	0.231	-0.074	**-0.712**	0.153	-0.094
15. Let me decide things for myself	0.406	-0.431	**0.568**	0.193	**-0.582**	0.546
16. Made me feel I wasn’t wanted	**-0.811**	-0.217	-0.131	**-0.602**	-0.487	-0.083
17. Could make me feel better when I was upset	**0.771**	0.065	-0.175	**0.805**	0.100	-0.135
18. Did not talk with me very much	**-0.869**	0.058	0.094	**0.812**	0.043	-0.020
19. Tried to make me feel dependent on her/him	-0.448	**0.596**	-0.288	-0.197	**0.759**	-0.148
20. Felt I could not look after myself unless she/he was around	-0.319	**0.682**	-0.276	-0.236	**0.714**	-0.144
21. Gave me as much freedom as I wanted	0.296	-0.443	**0.686**	-0.260	0.413	**0.664**
22. Let me go out as often as I wanted	-0.159	-0.207	**0.764**	-0.116	-0.277	**0.804**
23. Was overprotective of me	0.143	**0.789**	0.118	0.280	**0.778**	0.128
24. Did not praise me	**-0.814**	-0.171	-0.133	**-0.783**	-0.120	-0.111
25. Let me dress in any way I pleased	0.393	-0.301	**0.474**	0.075	-0.515	**0.553**

PBI–Parental Bonding Instrument; The highest value are bolded.

Table 2 shows the percentages of the variance explained. The three factors explain 64.12% of the cumulative variance in the PBI version of the mother and 63.40% of the cumulative variance in the PBI version of the father.

**Table 2:** Total explained variance for identified factors of Polish version of PBI (in group of adults, n = 473).

PBI Factors	For Mother version		For Father version	
	Proportion of variance (%)	Cumulative variance (%)	Proportion of variance (%)	Cumulative variance (%)
Factor 1- care	44.22	44.22	35.38	35.38
Factor 2- overprotection	14.55	58.77	22.33	57.71
Factor 3—autonomy	5.35	64.12	5.70	63.40

Table 3 shows the results of exploratory factor analysis in a group of adolescents. As in the adult group, here also the PBI showed a three-factor structure in both the mother and father versions. Item 6 in the mother version did not reach the assumed loading threshold for any of the factors. The highest loading index was achieved by items: item 12 in the mother’s version (Factor 1) and items 4 (Factor 1) and 22 (Factor 3). In turn, the weakest charging factors were obtained by items: item 13 in the mother’s version (Factor 3) and item 7 in the father’s version (Factor 2).

**Table 3:** Factor loadings of the Polish version of the Parental Bonding Instrument–results of exploratory factor analysis in the group of adolescents (n = 225).

PBI Items	Factors					
	PBI Mother version			PBI Father version		
	Factor 1- care	Factor 2- overprotection	Factor 3- autonomy	Factor 1- care	Factor 2- overprotection	Factor 3- autonomy
1. Spoke to me in a warm and friendly voice	**0.840**	-0.274	-0.090	**0.757**	-0.247	-0.284
2. Did not help me as much as I needed	**-0.776**	-0.192	0.078	**-0.689**	-0.242	0.083
3. Let me do those things I liked doing	0.080	-0.056	**0.743**	0.058	0.133	**0.795**
4. Seemed emotionally cold to me	**-0.878**	0.229	-0.060	**-0.805**	0.273	-0.164
5. Appeared to understand my problems and worries	**0.736**	-0.088	0.296	**0.746**	-0.096	0.219
6. Was affectionate to me	0.000	0.319	0.102	0.071	**0.568**	0.097
7. Liked me to make my own decisions	**0.487**	-0.208	0.334	0.348	**-0.501**	0.477
8. Did not want me to grow up	-0.321	**0.608**	-0.019	-0.156	**0.682**	-0.160
9. Tried to control everything I did	-0.168	**0.637**	-0.369	-0.322	**0.566**	-0.489
10. Invaded my privacy	-0.402	**0.507**	-0.323	-0.327	**0.555**	-0.478
11. Enjoyed talking things over with me	**0.842**	0.186	0.185	**0.764**	0.073	0.270
12. Frequently smiled at me	**0.896**	0.215	-0.115	**0.758**	0.150	-0.271
13. Tended to baby me	0.015	0.350	**-0.432**	-0.143	**0.600**	-0.100
14. Did not seem to understand what I needed or wanted	**-0.571**	0.164	-0.271	**-0.716**	0.202	-0.074
15. Let me decide things for myself	**0.585**	-0.363	0.331	0.285	-0.461	**0.597**
16. Made me feel I wasn’t wanted	**-0.841**	-0.245	-0.088	**-0.682**	-0.481	-0.132
17. Could make me feel better when I was upset	**0.708**	0.039	-0.333	**0.700**	0.095	-0.175
18. Did not talk with me very much	**-0.824**	0.198	0.090	**-0.726**	0.187	0.021
19. Tried to make me feel dependent on her/him	-0.434	**0.620**	-0.019	-0.258	**0.645**	-0.370
20. Felt I could not look after myself unless she/he was around	-0.416	**0.656**	-0.016	-0.316	**0.608**	-0.343
21. Gave me as much freedom as I wanted	0.396	-0.240	**0.685**	0.312	-0.176	**0.760**
22. Let me go out as often as I wanted	-0.259	-0.233	**0.690**	-0.160	-0.164	**0.802**
23. Was overprotective of me	0.068	**0.722**	0.216	-0.064	**0.745**	0.272
24. Did not praise me	**-0.844**	-0.197	-0.127	**-0.744**	-0.379	-0.199
25. Let me dress in any way I pleased	**0.599**	-0.473	0.125	0.296	**-0.553**	0.515

Note: PBI–Parental Bonding Instrument; The highest value are bolded.

Table 4 shows the percentages of the explained variance for PBI in the youth group. The three factories explain 60.00% of the cumulative variance in the PBI version of the mother and 61.62% of the cumulative variance in the PBI version of the father.

**Table 4:** Total explained variance for identified factors of Polish version of PBI (in group of adolescents, n = 225).

	For Mother version		For Fother version	
PBI Factors	Proportion of variance (%)	Cumulative variance (%)	Proportion of variance (%)	Cumulative variance (%)
Factor 1- care	46.19	46.19	44.19	44.19
Factor 2- overprotection	8.35	54.54	11.45	55.65
Factor 3- autonomy	5.46	60.00	5.97	61.62

### Results of test-retest corrrelation in group of adults

The above Table 5 shows the weakest stability obtained in the Factor 1 range, while Factors 2 and 3 show high stability over time.

**Table 5:** 3 weeks test–retest correlation for the factors of mater and father PBI version (retest for mothers n = 116; retest for fathers n = 112).

TEST–RETEST (after 3 weeks)	PBI Factors		
	Factor 1	Factor 2	Factor 3
	(Care)	(Overprotection)	(Autonomy)
Test-retest correlation for mother version (n = 116)	0.524***	0.869***	0.826***
Test-retest correlation for father version (n = 112)	0.581***	0.811***	0.880***

Note: PBI–Parental Bonding Instrument;

*p < 0.05;

***p < 0.001.

### Results of correlation analysis for PBI and Retrospective Assessment of Parents’ Attitudes Inventory

**Fig 1:**
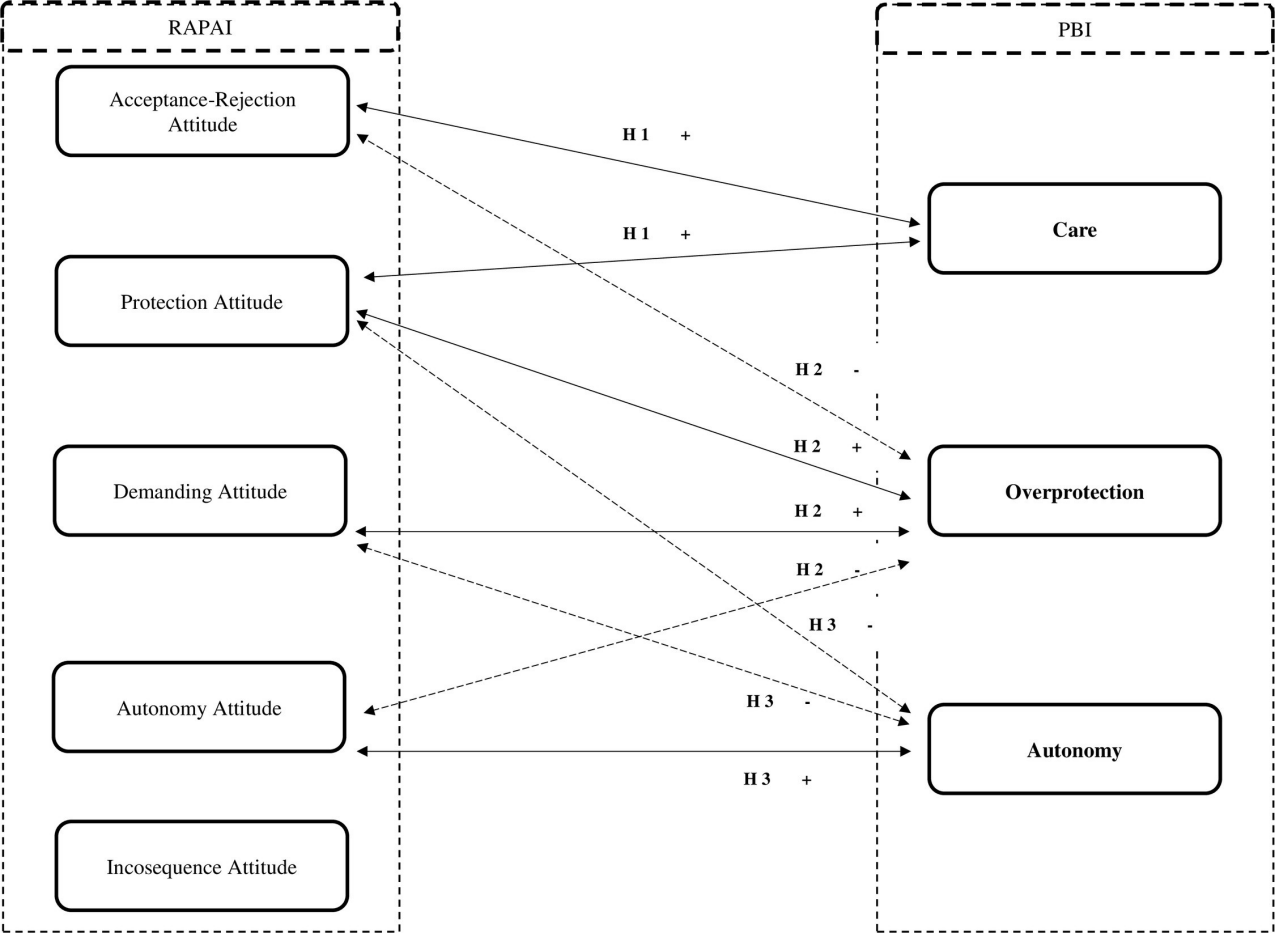
Model of studied hypotheses. Note: PBI–Parental Bonding Instrument; RAPAI: Retrospective Assessment of Parents’ Attitudes Inventory.

As a result of exploratory factor analyzes, it turned out that PBI in Polish version obtained a three-dimensional structure (Factor 1: Care; Factor 2: Overprotection; Factor 3: Autonomy). Next we used Retrospective Assessment of Parents’ Attitudes Inventory (RAPAI) in group of adoults to test the criterion validity of PBI, previously made the following hypotheses:

H1. The PBI Care dimension will positively correlate with the dimensions of Acceptance-Rejection Attitude (high) and Protection Attitude (average) in RAPAI;
H2. The PBI Overprotection dimension will correlate positively with the Demanding Attitude and Protection Attitude dimensions and negatively with the Autonomy Attitude and Acceptance-Rejection Attitude dimensions in RAPAI;
H3. The PBI Autonomy dimension will correlate positively with the Autonomy Attitude dimension and negatively with the Demanding Attitude and Protection Attitude dimensions in RAPAI. Fig 1 below shows a hypothetical model of the correlation between Parental Bonding Instrument and Retrospective Assessment of Parents’ Attitudes Inventory.

**Fig 2:**
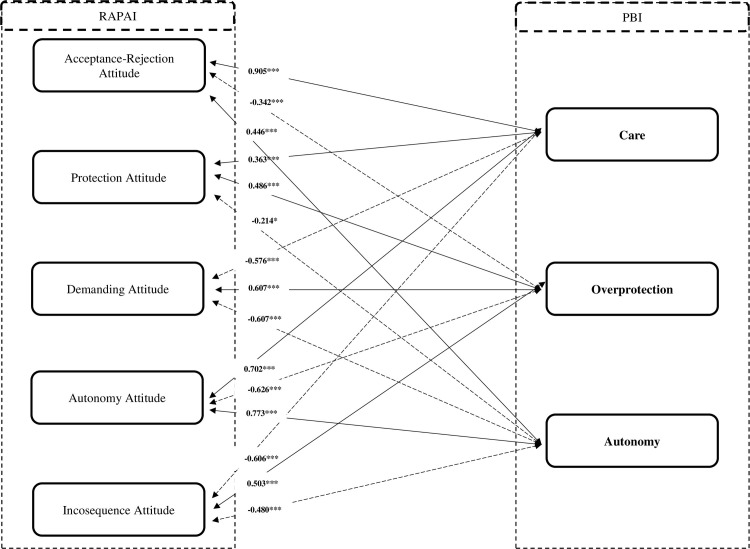
Correlation analysis for PBI dimensions and RAPAI dimensions for mother. Note: PBI–Parental Bonding Instrument; RAPAI: Retrospective Assessment of Parents’ Attitudes Inventory; *p < 0.05; ***p < 0.001.

**Fig 3:**
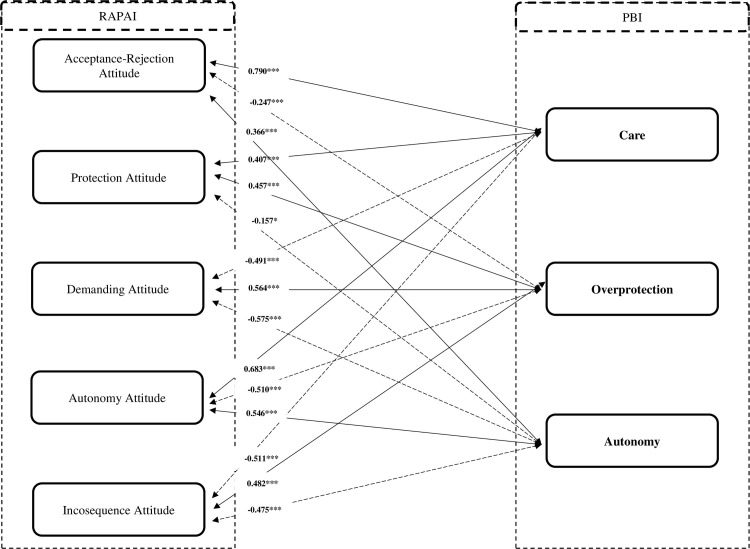
Correlation analysis for PBI dimensions and RAPAI dimensions for father. Note: PBI–Parental Bonding Instrument; RAPAI: Retrospective Assessment of Parents’ Attitudes Inventory; *p < 0.05; ***p < 0.001.

Figs 2 and 3 present the results of Pearson’s r correlation analysis between PBI and Retrospective Assessment of Parents’ Attitudes Inventory (RAPAI). The previously presented hypotheses were confirmed like:

H1. The PBI Care dimension positively and significant (p<0.001) correlates with the dimensions of Acceptance-Rejection Attitude (0.905 for mother version; 0.790 for father version) and Protection Attitude (0.363 for mother version; 0.407 for father version);
H2. The PBI Overprotection dimension positively correlates and significant (p<0.001) with the dimensions of Demanding Attitude (0.607 for mother version; 0.564 for father version) and Protection Attitude (0.486 for mother version; 0.457 for father version) and negatively with the dimensions of Autonomy Attitude (-0.626 for mother version; -0.510 for father version) and Acceptance-Rejection Attitude (-0.342 for mother version; -0.247 for father version);
H3. The PBI Autonomy dimension correlates positively and significant (p<0.001) with the Autonomy Attitude dimension (0.773 for mother version; 0.546 for father version) and negatively with the Demanding Attitude dimensions (-0.607 for mother version; -0.575 for father version) and Protection Attitude (-0.214 for mother version; - 0.157 for father version).

## Discussion

The article presents the factor analysis and psychometric properties of the Parental Bonding Instrument by Parker et al., [9] in the group of adults and in the youth group. Exploratory factor analyzes have shown that the Polish, 25-item version of the PBI adopts a three-factor structure in both groups (Tables 1 and 3).

In adults version of PBI, the composition of Factor 1, both in the version for mother and father, includes 12 items, the composition of Factor 2–8 items for mother version and 9 items for father version, and the composition of Factor 3–5 items for mother version and 4 items for mother version. Looking at what items were included in the individual components, the following names of factors can be proposed: "care", "overprotection", "autonomy". For the care factor, factor analysis showed the same items as for the original two-dimensional version of Parker et al., [9], and in other versions in which the three-factor structure was demonstrated among adults, e.g. in the Australian studies by Cubis et al. [48], in studies by Murphy et al., [37] among participants from the UK and the USA, in a study by Mohr et al., [39] in the French studies and in British studies [38]. As for the "overprotection" and "autonomy" factors, similar results were also obtained as in the above-cited. Studies in which a three-factor structure was obtained, where compared to the original version, 6 out of 13 items from the "overprotection" factor were included in the "autonomy" factor. Interesting results were obtained for the following items: 7 (Liked me to make my own decisions) and 15 (Let me decide things for myself)—which approximately load the second and third factors, while in the version for the mother, item 7 loads the second factor more strongly. and item 15 has a stronger third factor, while in the father’s version both factors load factor 2 more strongly.

An analysis of the content of these items shows that both are concerned with decision making. Therefore, issues related to shaping decision-making in the educational process may have a different meaning in mother-child and father-child relations. In other countries, there were also items that loaded more than one factor to a different degree. For example, in the British version [38], also item 7 (Liked me to make my own decisions) charged two factors—however, it was the first factor and the third factor for both father and mother, and the values were much lower. Various hypotheses as to why this is so can be put forward, including those related to cultural differences (e.g. the specificity of educational practices in Poland—general social beliefs—how much freedom and decisions should be left to the child). However, this issue requires more studies.

The factor structure study in the adolescent group also showed three factors It should be pointed out that item 6 in mother version (Was affectionate to me) in adolescent sample did not exceed the cut-off point assumed in the research, so it was not included in any factor. Hence, the mother version will eventually contain 24 items. In addition, item 6 in father version loads Factor 2 (overprotection), affectionate from father is perceived as overprotection—unlike in the adult study, where item 6 in both mother and father version loaded Factor 1 (care). One explanation for such an issue may concern the very understanding of the meaning of the content of the item. In the Greek version [62], already at the stage of translation, the authors changed the content to: "is loving towards me", it should be noted that children aged 11–14 years participated in these studies (average 11.94), and in our study slightly older 13–19 years (average 16 years). It is worth paying attention to item 13 ("Tended to me baby"), which in the adult study unambiguously loaded Factor 2 (overprotection), and in the youth study similarly in the version for the father (Factor 2), while for the mother it loaded Factor 3 (autonomy). Issues related to the translation itself as well as cultural differences may come into play. This item was translated without clearly distinguishing between baby/child. In Polish, the word "child" may be acceptable for young people—especially from the mother’s side—and this is how it has been translated. In the English version, the word "baby" can be translated into Polish as "very small child/infant".

The stability over time analysis, where a retest was performed after 3 weeks, showed mixed results. For Factors 2 and 3, both in the mother and father versions, a high correlation between the first and second measurement was noted, at a level above 0.8, which indicates high stability in this respect. Factor 1 showed relatively weaker stability, where in both versions, for mother and father, a correlation oscillating around 0.6 was noted. One possibility of a low Factor 1 (care) score is that it has 12 items, 5 of which are formulated in negative way. In Polish, it is very troublesome and may cause problems for the respondents, in particular when denying a negative statement (there is a phenomenon of double negation)[63, 64]. For comparison, it can be mentioned that there is only one negative item in Factor 2 (overprotection) and not one in Factor 3 (autonomy).

Following the exploratory factor analysis, criterion validity was also carried out through correlation with another tool known in Poland: Retrospective Assessment of Parents’ Attitudes Inventory by Plopa [60]. Three hypotheses were made and all three were confirmed. First, as assumed, the Care Dimension (PBI) for both the mother and the father correlated positively and particularly highly with the Acceptance-Rejection Attitude (RAPAI) dimension. This result means that the respondents who obtained high scores in Factor 1 (Care) in PBI also declared a high level of acceptance and sense of closeness with their parents (according to RAPAI). This confirms the assumptions about the Care in PBI. Secondly, the PBI Overprotection dimension positively correlated with the Demanding Attitude and Protection Attitude dimensions, although in the case of the latter one could suspect a higher correlation, because high scores in these RAPAI dimensions mean excessive parental control and demands on the child. In the other hand, as we assumed, Factor 2 PBI (Overprotection) negatively correlated with the dimensions of Autonomy Attitude and Acceptance-Rejection Attitude, which means that parents perceived as overprotective in PBI gave less autonomy and were more overwhelming according to the respondents. Third and finally, Factor 3 PBI (Autonomy) correlated positively with the Autonomy Attitude dimension and negatively with the Demanding Attitude and Protection Attitude dimensions. Especially, high correlation in in Autonomy in both tools confirms the importance of distinguishing this factor in PBI. The Retrospective Assessment of Parents’ Attitudes Inventory contains one more dimension that was not included in the hypotheses—Inconsequence Attitude. The correlation between PBI and RAPAI shows that Inconsequence Attitude negatively correlates with Factor 1 (Care) and Factor 3 (Autonomy), and positively with Factor 2 (Overprotection) in PBI—these results also confirms the assumptions of the PBI as regards the examined parental attitudes. All the above-described correlations were statistically significant at the level of p <0.001, with the exception of Autonomy in PBI and Protection Attitude in RAPAI, where the statistical significance was p <0.05

The research presented in this article has its limitations. In particular, it should be taken into account that the study group was heterogeneous—among adults it consisted of a combined clinical and non-clinical sample, while the youth group was significantly smaller, with a large majority of girls. Further studies should also include the possibility of linguistic correction of some items, and even changing negative items into positive items—as in many other studies (e.g. [38, 41]). Moreover, it would be advisable to explore the possibility of using the 16-item version proposed by Kendler et al. [47].

## Conclusions

The Polish, 25-item version of the PBI is characterized by a three-factor structure, both in the adult and adolescent study. In the study of adolescents in the mother version, one of the items (item 6) loaded factors below the adopted cut-off point. In the adult and adolescent study, high reliability was found for almost all factors ranging from 0.816 to 0.948, with the exception of the maternal version of Factor 3 in the adolescent study, which took a lower but acceptable value. Therefore, it should be considered that in polish version of PBI factors loaded differently. It refers to factors of adults compared to adolescents, and the weaker reliability of Factor 3 among adolescents in the mother version. Stability of PBI over time (study 3 weeks apart) showed high stability in terms of factors 2 and 3 and moderate stability in terms of factor 1. Discussion of the results and comparison with studies in other countries revealed that when using PBI especially among adolescents, one should be sensitized to additional language comprehension issues. The Polish version of the PBI also showed satisfactory criteria accuracy. To our knowledge, it is the first adaptation of PBI in Poland that can be useful in both research and clinical practice.
